# Diagnostic Performance, Triage Safety, and Usability of a Clinical Decision Support System Within a University Hospital Emergency Department: Algorithm Performance and Usability Study

**DOI:** 10.2196/46760

**Published:** 2023-08-31

**Authors:** Juhani Määttä, Rony Lindell, Nick Hayward, Susanna Martikainen, Katri Honkanen, Matias Inkala, Petteri Hirvonen, Tero J Martikainen

**Affiliations:** 1 Klinik Healthcare Solutions Oy, Helsinki, Finland; 2 Department of Health and Social Management, University of Eastern Finland, Kuopio, Finland; 3 Department of Emergency Care, Kuopio University Hospital, Kuopio, Finland

**Keywords:** clinical decision support system, emergency department, performance, usability, user experience, validation, medical care, decision-making, digital health, differential diagnosis, triage, patient population

## Abstract

**Background:**

Computerized clinical decision support systems (CDSSs) are increasingly adopted in health care to optimize resources and streamline patient flow. However, they often lack scientific validation against standard medical care.

**Objective:**

The purpose of this study was to assess the performance, safety, and usability of a CDSS in a university hospital emergency department setting in Kuopio, Finland.

**Methods:**

Patients entering the emergency department were asked to voluntarily participate in this study. Patients aged 17 years or younger, patients with cognitive impairments, and patients who entered the unit in an ambulance or with the need for immediate care were excluded. Patients completed the CDSS web-based form and usability questionnaire when waiting for the triage nurse’s evaluation. The CDSS data were anonymized and did not affect the patients’ usual evaluation or treatment. Retrospectively, 2 medical doctors evaluated the urgency of each patient’s condition by using the triage nurse’s information, and urgent and nonurgent groups were created. The *International Statistical Classification of Diseases, Tenth Revision* diagnoses were collected from the electronic health records. Usability was assessed by using a positive version of the System Usability Scale questionnaire.

**Results:**

In total, our analyses included 248 patients. Regarding urgency, the mean sensitivities were 85% and 19%, respectively, for urgent and nonurgent cases when assessing the performance of CDSS evaluations in comparison to that of physicians. The mean sensitivities were 85% and 35%, respectively, when comparing the evaluations between the two physicians. Our CDSS did not miss any cases that were evaluated to be emergencies by physicians; thus, all emergency cases evaluated by physicians were evaluated as either urgent cases or emergency cases by the CDSS. In differential diagnosis, the CDSS had an exact match accuracy of 45.5% (97/213). The usability was good, with a mean System Usability Scale score of 78.2 (SD 16.8).

**Conclusions:**

In a university hospital emergency department setting with a large real-world population, our CDSS was found to be equally as sensitive in urgent patient cases as physicians and was found to have an acceptable differential diagnosis accuracy, with good usability. These results suggest that this CDSS can be safely assessed further in a real-world setting. A CDSS could accelerate triage by providing patient-provided data in advance of patients’ initial consultations and categorize patient cases as urgent and nonurgent cases upon patients' arrival to the emergency department.

## Introduction

Digital health technology is increasingly being developed to support health care systems around the world. Digital health technology includes solutions that assess the urgency and the differential diagnosis of the patient’s symptoms with the help of artificial intelligence. These solutions are frequently called *clinical decision support systems* (CDSSs) or *computerized diagnostic decision support programs* [1,2].

These systems can either aid health care professionals in their decision-making or give information on symptoms, conditions, and possible recommendations for future actions to the patient. However, patient-provided data solutions are rarely scientifically validated [3,4]. There are very few studies that have assessed solution performances in real-world settings, and these are rarely performed using a patient sample with a broad range of conditions [3,5-8].

Work in triage and emergency departments is highly demanding, with substantial time pressure. There is a risk of human errors when operating with high patient volumes, acute conditions, and severe stress. Physicians have been estimated to have a 5% diagnostic error rate [9], with half of these errors being potentially harmful [10]. In an emergency department setting, a CDSS based on patient-provided data that are shared prior to patients entering the emergency department could have the potential to significantly help in allocating optimal resources for the patients who need more prompt assessments and complex care.

The extensive need for the validation of any CDSS with patient-provided data has been acknowledged widely [2,4,5,7]. Studies that evaluate the accuracy or diagnostic performance of a CDSS with patient-provided data in a wide and diverse set of patients are rare and have mainly used clinical vignettes rather than actual patients in a real-life setting [3,4]. A clinical vignette study can describe the experimental accuracy and performance of a system algorithm, but it (1) omits the complex diversities and randomness among patients in real life, (2) typically concentrates only on textbook cases, and (3) omits the usability of the system. Combined clinical vignette studies mainly describe the theoretical performance of an algorithm and do not validate the performance of the CDSS in actual use. Hence, a CDSS with patient-provided data should always take usability into consideration. High usability is one of the key factors that allow a system to make correct interpretations when dealing with patient-provided data. The system needs to be widely easy to use and effective (ie, usable) [11]. One of the most popular questionnaires for assessing system usability is the System Usability Scale (SUS) questionnaire [12-14]. A positive version of the SUS questionnaire has, in addition, been developed [13].

The aim of this study was to analyze the performance of a particular CDSS (Klinik Access [Klinik Healthcare Solutions Oy]) with patient-provided data in a real-world university hospital emergency department setting. The main aims were to (1) evaluate the performance of the system in detecting the clinical urgency of a patient’s condition and (2) evaluate diagnostic performance by using the actual *International Statistical Classification of Diseases, Tenth Revision* (*ICD-10*) diagnoses assigned at the emergency department. Our third aim was to assess the usability of the system in a real-world setting with real patients. Our hypotheses were that the CDSS has acceptable safety margins and sensitivity in clinically urgent cases, diagnostic performance correlates well with actual medical diagnoses, and the usability of the system is good or better.

## Methods

### Study Population

The study population consisted of patients who entered the emergency department of Kuopio University Hospital, Finland, during a 3-week period in September 2020. The patients were recruited by the research assistants in the emergency department waiting room while waiting for a triage nurse’s assessment. Participation in this study was fully voluntary. The following patients were excluded from this study: patients aged 17 years or younger, patients with cognitive impairments, and patients who entered the unit in an ambulance or with the need for immediate care. The patients were informed about this study, including the information that this study would not affect their emergency department visit or received treatment in any way. To verify their acceptance to participate in this study, the patients provided written informed consent.

### Ethics Approval

This prospective study has been reported in ClinicalTrials.gov (NCT04577079), and this study was approved by the research ethics committee of the Northern Savo Hospital District (347/2020), Finland.

### Data Collection, Urgency Evaluation, and Diagnosis

After consenting to study participation, patients completed the CDSS (Klinik Access) web-based form independently, using tablet computers that were provided by the research assistants. The form was used to obtain information, including demographics, history, and symptom-related factors. The data provided by the patients were not used by the emergency department personnel. After the data collection phase, 2 independent physicians (KH, MD [15 y of experience]; MI, MD [7 y of experience]) assessed the patient data from the electronic health records. The assessing physicians did not have any access to or knowledge of the CDSS data.

First, the physicians assessed the urgency of each patient case, using only written information provided by the triage nurse—the identical information on which urgency is based in the emergency department. Urgency was mapped to the same four categories that the CDSS software used (Table 1). After evaluating the urgency, the assessing physicians reviewed the visits in the electronic health records and checked that the correct *ICD-10* diagnoses were provided by the emergency department clinicians.

**Table 1.** Evaluation of the urgency of a patient’s condition and clinical examples of related conditions.

Urgency	Definition	Clinical example (symptom or condition)
Self-care	Benign condition that could primarily be self-treated by the patient (ie, no real need for assessment by a clinician)	Mild viral infection Hives (urticaria)
Nonurgent	Condition that requires nonurgent evaluation by a clinician (ie, patient should primarily receive a nonurgent [general practitioner] appointment)	Prolonged cough in an otherwise healthy young patient Symptomatic knee arthrosis without significant disability
Urgent	Condition that requires evaluation by a clinician within the next few days, preferably during the same day	Ear pain with only temporary relief via analgesics Severe shoulder pain from an injury
Emergency	Condition that should be evaluated by a clinician within 2 hours or as soon as possible	Chest pain Breathing difficulty

### The CDSS

**Figure 1.**
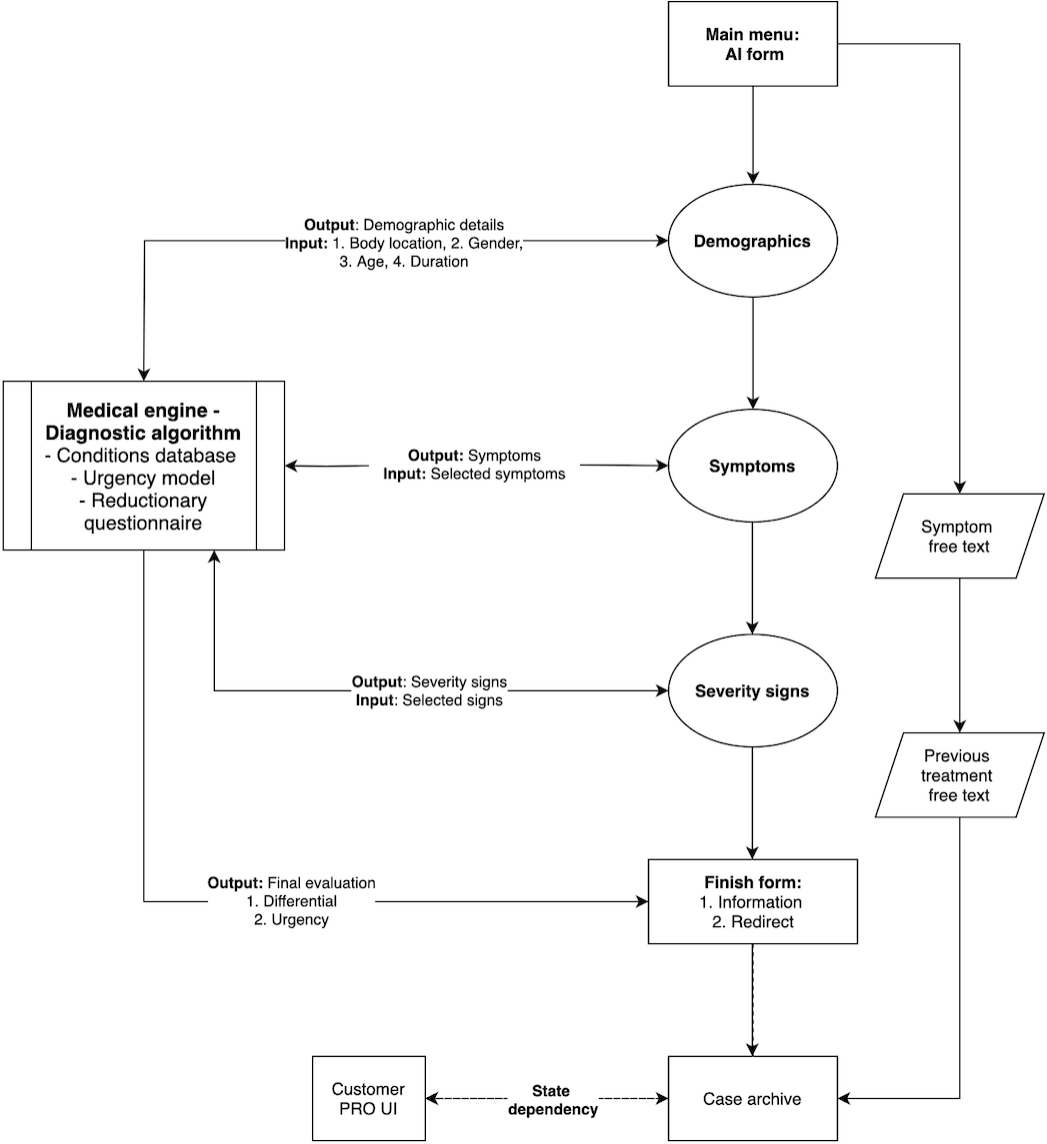
The system process of our computerized clinical decision support system (Klinik Access). In this study, “finish form information” was the request to complete the positive version of the System Usability Scale questionnaire, after which the patients were directed to wait for the triage nurse to call them in. Customer PRO UI was not used in this study, and case information was only archived for the study purposes. AI: artificial intelligence; Customer PRO UI: Customer Professional Interface.

The CDSS used in this study was Klinik Access (version 1.1). The system intends to support primary care health care organizations by receiving service inquiries from patients, automatically preanalyzing the included clinical information, and supporting health care professionals in managing the triage demand effectively. It has already been implemented in over 400 primary care and dental facilities in the Nordics, the United Kingdom, and the Netherlands. Klinik Access evaluates patient symptoms and potential conditions by using the situational data provided by the patients (Figure 1). It consists of an interface for receiving the patient inputs, a back end for generating the computational dynamic questionnaire and other business logic, and a professional interface for the management of the patient inquiries by health care staff. It was originally developed for general primary care use, including children and adolescent care use. Although it properly recognizes various urgent and emergency situations, it has not yet been optimized for use solely in an emergency department context. As such, the clinical context in this study was intended to be partially experimental.

The Klinik Access system uses a medical gold standard Bayesian methodology [15,16] for inferring the effect of clinical features on the probabilities of the differential diagnosis conditions. The severity and the urgency of a condition were inferred, in addition to the differential diagnosis, by using specific severity symptoms and by setting a threshold (15%) for the probabilities of relevant conditions in the differential. The purpose of this study was to analyze the performance of the algorithm and the patient usability of the CDSS. As such, only the system output data were analyzed after data collection, and the professional interface was not used or assessed in this study.

### Usability

Usability was evaluated by using the positive version of the SUS questionnaire [13]. The questionnaire has 10 items that are rated on a 5-point Likert scale. The difference between the original and positive versions of the questionnaire is that the positive version has only positive statements about usability, whereas in the original one, every other statement is negative [13]. The positive version of the SUS questionnaire was used, as respondents and researchers are less likely to make mistakes when filling in and analyzing the questionnaire, respectively [13]. After completing the web-based CDSS form, the patients filled in the positive version of the SUS questionnaire, and they had the opportunity to give free-text feedback by using the web-based form.

### Statistical Analysis

The estimated required sample size for this study (estimated via a power analysis) was a minimum of 246 patients (confidence level=95%; margin of error=5%; study population proportion=80%). A patient was excluded from the analyses if they had intentionally ignored filling in the majority of the web-based form, misused the form, or stated that they were not willing to participate in this study in the free-text field. The sensitivity and positive predictive values, with 95% CIs, were calculated for the urgency evaluations between the CDSS and the two assessing physicians, and the mean of these results was used to report the performance of the CDSS in comparison to that of the assessing physicians’ evaluations. When evaluating the performance of the assessing physicians, the sensitivity was calculated for both assessors, and the mean was used to report the performance of the assessors.

The urgency of the patients’ needs was evaluated, using 4 categories, by the CDSS and the assessors. However, as the patients were practically grouped only into 2 groups (nonurgent and urgent) in the emergency department, we dichotomized the urgency as nonurgent (blue=0; green=1) and urgent (yellow=2; red=3) cases for the analyses.

The diagnostic accuracy of the CDSS was evaluated, using the differential diagnosis proposed by the system. A condition had to exceed the fixed probability threshold of 15% to be included in the differential diagnosis list. The diagnostic match was evaluated as “exact” if the actual *ICD-10* diagnosis from electronic health records was included in the differential diagnosis list proposed by the CDSS. The diagnostic match was evaluated as “close” if the condition proposed by the CDSS in the differential diagnosis list was a close match to the actual *ICD-10*–coded diagnosis (Table 2). The statistical software IBM SPSS Statistics version 25 (IBM Corporation) and Microsoft Excel 2022 (Microsoft Corporation) were used.

**Table 2.** The prevalence of the most frequent *International Statistical Classification of Diseases, Tenth Revision* diagnoses from the electronic health records of the whole study population and the prevalence of the most frequent diagnoses with exact and close matches to those produced by the clinical decision support system (CDSS).

Diagnosis		Frequency
**Diagnoses from electronic health records, n (%)**		
	A46 Erysipelas	8 (3.8)
	M54.5 Low back pain	7 (3.3)
	M54.4 Lumbago with sciatica	6 (2.8)
	M79.6 Pain in limb, hand, foot, fingers, and toes	6 (2.8)
	R10.3 Pain localized to other parts of lower abdomen	6 (2.8)
	R10.4 Other and unspecified abdominal pain	6 (2.8)
	K35.9 Acute appendicitis, unspecified	5 (2.3)
	T81.4 Infection following a procedure	5 (2.3)
	R07.4 Chest pain, unspecified	4 (1.9)
	S61.0 Open wound of finger(s) without damage to nail	4 (1.9)
	H16.9 Keratitis, unspecified	3 (1.4)
	I49.9 Cardiac arrhythmia, unspecified	3 (1.4)
	J06.9 Acute upper respiratory infection, unspecified	3 (1.4)
	R06.0 Dyspnea	3 (1.4)
	R10.1 Pain localized to upper abdomen	3 (1.4)
	S52.5 Fracture of lower end of radius	3 (1.4)
	S93.4 Sprain and strain of ankle	3 (1.4)
	T15.0 Foreign body in cornea	3 (1.4)
**Diagnoses with exact matches to the CDSS, n**		
	R10.3 Pain localized to other parts of lower abdomen	6
	A46 Erysipelas	5
	M54.5 Low back pain	5
	R10.4 Other and unspecified abdominal pain	5
	M79.6 Pain in limb, hand, foot, fingers, and toes	4
	S61.0 Open wound of finger(s) without damage to nail	4
	H16.9 Keratitis, unspecified	3
**Diagnoses with close matches to the CDSS, n**		
	M54.4 Lumbago with sciatica	3

## Results

### Study Population Characteristics

There were 259 patients who had completed the CDSS form and had comparable information in the electronic health records. Of these patients, 11 were excluded (4 patients were aged younger than 18 y, 5 patients had an emergency department visit that was solely associated with an earlier emergency department or control visit, and 2 patients had an inadequately filled form). Thus, there were 248 patients in total, with 122 (49.2%) female patients and 126 (50.8%) male patients, and the mean age was 46 (range 18-82) years. The median number of symptoms provided by patients to the CDSS form was 5 (range 1-20).

### Urgency

Physician A evaluated all 248 patient cases. However, physician B missed 12 patient cases due to difficulties in accessing appropriate electronic health record data, thus leaving 236 comparable patient cases in total (Tables 3-6). The mean sensitivities were 85% for urgent cases and 19% for nonurgent cases when assessing the performance of the CDSS evaluations in comparison to those of the physicians. The corresponding mean sensitivities were 85% and 35%, respectively, when comparing the evaluations of the assessing physicians. The CDSS did not miss any cases that were evaluated to be emergencies by physicians, that is, all emergency cases evaluated by the physicians were evaluated as either urgent cases or emergency cases by the CDSS (Tables 3-6).

**Table 3.** The urgency evaluations of the computerized decision support system (CDSS) and physician A (cases: n=248).^a^

		Physician A cases, n			
		Self-care	Nonurgent	Urgent	Emergency
**CDSS cases, n**					
	Self-care	3	4	5	0
	Nonurgent	2	4	23	0
	Urgent	2	32	97	4
	Emergency	0	14	54	4

a The positive predictive values for nonurgent and emergency cases were 32% (95% CI 20%-46%) and 77% (95% CI 74%-79%), respectively. The sensitivity values for nonurgent and emergency cases were 21% (95% CI 12%-34%) and 85% (95% CI 79%-90%), respectively.

**Table 4.** The urgency evaluations of the computerized decision support system (CDSS) and physician B (cases: n=236).^a^

		Physician B cases, n			
		Self-care	Nonurgent	Urgent	Emergency
**CDSS cases, n**					
	Self-care	0	4	8	0
	Nonurgent	0	1	25	0
	Urgent	0	17	107	5
	Emergency	0	18	55	6

a The positive predictive values for nonurgent and emergency cases were 13% (95% CI 6%-26%) and 87% (95% CI 85%-89%), respectively. The sensitivity values for nonurgent and emergency cases were 17% (95% CI 6%-35%) and 84% (95% CI 78%-89%), respectively.

**Table 5.** The urgency evaluations of physician A and physician B (cases: n=236; physician A vs physician B).^a^

		Physician B cases, n			
		Self-care	Nonurgent	Urgent	Emergency
**Physician A cases, n**					
	Self-care	0	4	3	0
	Nonurgent	0	10	42	0
	Urgent	0	16	147	6
	Emergency	0	0	3	5

a The sensitivity values for nonurgent and emergency cases were 47% (95% CI 28%-66%) and 78% (95% CI 72%-84%), respectively.

**Table 6.** The urgency evaluations of physician A and physician B (cases: n=236; physician B vs physician A).^a^

		Physician A cases, n			
		Self-care	Nonurgent	Urgent	Emergency
**Physician B cases, n**					
	Self-care	0	0	0	0
	Nonurgent	4	10	16	0
	Urgent	3	42	147	3
	Emergency	0	0	6	5

a The sensitivity values for nonurgent and emergency cases were 24% (95% CI 14%-37%) and 91% (95% CI 86%-95%), respectively.

### Differential Diagnosis

Of 248 patients, 35 had to be excluded from the differential diagnosis analyses due to the absence of an *ICD-10* diagnosis in the electronic health records or for having a code denoting a Z-diagnosis (factors influencing health status or contact with health services). Thus, 213 cases in total were included in the differential diagnosis analyses. The results of the differential diagnoses of the CDSS are shown in Table 7. The CDSS had an exact match accuracy of 45.5% (97/213), with an additional close match in 12.7% (27/213) of patients. The most frequent actual diagnoses from the electronic health records, including exact- and close-match diagnoses, are presented in Table 2. Other close-match diagnosis examples are H43.8 (other disorders of the vitreous body; CDSS suggestion: visual disturbances), K64.0 (first-degree hemorrhoids; CDSS suggestion: bleeding from anus), and H16.0 (corneal ulcer; CDSS suggestion: foreign object in the eye). The median number of diagnoses (ie, conditions) within the differentials provided by our CDSS was 5 (range 0-21).

**Table 7.** The results of the differential diagnostics of the clinical decision support system (CDSS), including possible explanatory factors for missed diagnoses.

Differential diagnosis		CDSS accuracy (diagnoses: N=213), n (%)
Exact match		97 (45.5)
Close match		27 (12.7)
**Missed diagnoses**		89 (41.8)
	False location	9 (4.2)
	Inadequate response	25 (11.7)
	No identified diagnosis^a^	16 (7.5)
	Other miss	39 (18.3)

a The exact *International Statistical Classification of Diseases, Tenth Revision* diagnosis was not included in the CDSS differential diagnostics selection.

### Usability

A positive version of the SUS questionnaire was answered by 95.4% (247/259) of the whole study population. The mean SUS score for the CDSS was 78.2 (SD 16.8). A total of 31 patients had given feedback on the CDSS, of whom 12 gave positive feedback, 14 had some critiques or suggested certain changes, and 5 gave neutral feedback. The most frequent free-text feedback for improving the CDSS addressed possible challenges for older patients (n=5) and the need for guidance to fill in the form (n=3).

## Discussion

### Overview

To the authors’ knowledge, this is the first real-world study that uses a large population with a wide age range to extensively evaluate the performance and usability of a CDSS for urgency evaluation and differential diagnosis in a patient setting. The CDSS was found to be equally as sensitive as the assessing physicians in terms of urgent patient evaluation, but the CDSS underperformed in nonurgent patient cases. The CDSS was considered to be safe, as none of the emergency cases evaluated by physicians were evaluated as nonurgent cases by the CDSS. The diagnostic performance of the CDSS was good, with a 45.5% (97/213) exact match accuracy and 12.7% (27/213) close match accuracy for differential diagnosis in a real-world setting, and it included a vast range of possible diagnoses. The usability of the CDSS was considered to be impressive, with a mean SUS score of 78.2 (SD 16.8).

Digital health technologies and CDSSs have the potential to optimize health care resources and enable patients to manage their conditions themselves more effectively [4]. In addition, CDSS technologies have been suggested to have a significant role in helping with both patient management and triage [17]. Digital health technologies, including CDSSs, are however often lacking an evidence base [18]. Both the importance and the lack of validation for digital health technologies have been acknowledged widely [2,19].

With web-based CDSS access, patients can complete their inquiries at home or prior to their emergency department arrival. Health care and triage personnel would then have comprehensive written information on the patient’s symptoms, demographics, and history data available when the patient enters the emergency department. This can help to speed up the work in triage considerably. A CDSS could also categorize patient cases as urgent and nonurgent cases when patients enter the emergency department. This would improve the waiting times for patients with urgent conditions and thus improve treatment outcomes [1].

Even though any CDSS should not be too risk averse [4], it is of utmost importance that the CDSS is safe enough to avoid missing any emergency cases. Considering the high resource demands and massive time pressures within the emergency department, directing even every fifth patient safely to nonurgent care would be very beneficial. This is a significant factor to consider for the patient population who would, in any case, enter the emergency department, regardless of possible overtriaging. Eventually, the purpose of a CDSS in the emergency department is to relieve the health care personnel’s burden, so that time and effort can be optimally allocated [20,21]. Additionally, to fully gain the benefits of a CDSS, it is important that the CDSS is properly implemented in health care professionals’ everyday work and workflow [22].

### Urgency Evaluation

In this study, the CDSS was found to be as sensitive as physicians in identifying urgent patient cases. For nonurgent patient cases, the CDSS was oversensitive. This is logical when considering the nature of artificial intelligence–driven digital health technology in the health care field, where safety is of utmost importance. As described previously, there is a great need to validate every CDSS in real-world clinical settings [4,7].

The original purpose of this study was to assess the triage nurses’ assessments, using the Emergency Severity Index (ESI) [23]. However, the distribution of the ESI scores was greatly skewed; no patients had an ESI score of 1, and only 2% (5/248) of patients had an ESI score of 2, which substantially limited data analysis. Therefore, the physicians were considered to be the most suited to assessing the urgency of the patients’ conditions by using the triage nurse’s information.

There is a lack of studies assessing CDSSs for urgency evaluation in a real-world setting with a broad range of possible conditions [7,8]. Cotte et al [24] assessed the triage performance of a CDSS (Ada) in a real-world emergency department setting with 344 patients. They found overtriage in 57% of cases and undertriage in 9% of cases when the CDSS was compared to physicians’ evaluations. Yu et al [25] assessed 2 CDSSs (ie, symptom checkers) retrospectively for 100 real-world patients that entered the emergency department, and they found a sensitivity of 58%; however, this study was performed retrospectively by researchers and thus lacked an acute emergency department setting and patient usability evaluation. Schmieding et al [26] assessed the performance of 22 CDSS technologies in 2015 and 2020 and discovered no improvement in triage performance over the 5-year period. However, to conclude, studies assessing CDSS performance underline the importance of real-world data. This is because clinical vignettes have been found to have considerable inherent limitations when used to assess diagnostic accuracy or triage safety, in comparison to real-world data [5]. Just recently, Fraser et al [8] evaluated the performance and usability of a CDSS (Ada; ie, in a similar setting to ours) in an emergency department. In terms of urgency, among 37 patient cases, 22% and 14% were considered unsafe and too risky by at least one or two out of three physicians, respectively [8].

### Differential Diagnosis

When assessing the CDSS’s diagnostic performance with *ICD-10* diagnoses set by physicians in the patients’ electronic health records, an exact diagnostic match was found in 45.5% (97/213) of cases, and missed diagnoses were found in 41.8% (89/213) of cases. Given the real-world, acute emergency department study setting, this can be considered a good performance. As described previously, studies assessing the performance of a CDSS usually use vignettes without real patient data, which makes it difficult to draw any clinical conclusions [4,5]. In a review by Wallace et al [7], there were only a few studies that assessed diagnostic accuracy by using real patient data, and this was done only in specific subspecialties. Despite these facts, the accuracy of the primary diagnosis was found to be low, with a range of 19% to 38% [7]. In a recent emergency department study that used another CDSS, the sensitivity of the differential diagnosis was far higher (70%) [8].

This study assessed the exact and close diagnostic matches between our CDSS and electronic health record diagnoses. Through this, we wanted to highlight the challenges faced when using real-world data. Even though the CDSS evaluated in this study contained over 500 medical conditions, patients may experience rare conditions that are difficult and often futile to include within CDSS differential diagnostic algorithms. Among 213 patients, there were 16 patients (7.5%) who had an *ICD-10* diagnosis that was not included in the CDSS differential diagnostics, such as myelodysplastic syndrome (D46.9). As described previously, there are numerous diagnoses that may not be necessary to include in CDSS differential diagnostics. This is because some diagnoses may be too niche for emergency department triage or are only relevant for certain specialist clinical settings, such as tertiary subspecialty care.

The main objective of any CDSS is to provide support for triage evaluation, as setting an accurate diagnosis often requires specific tests, such as blood sample and imaging tests [7]. Therefore, it is impractical to aim for the perfect diagnostic performance of a CDSS. Nevertheless, a CDSS could optimally guide health care professionals to consider a patient’s relevant differential diagnosis.

Unlike several CDSSs, the one used in this study (Klinik Access) proposed a differential diagnostic list by setting a threshold (15%) for the probabilities of relevant conditions. This mimics the testing threshold design in a physician’s clinical decision-making, during which all of the relevant and possible conditions are considered [27,28].

With regard to practical issues, there are multiple factors that affect a unique patient case in real life and are complicated to include in theoretical studies with clinical vignettes. For instance, in this study, some patients were referred to the emergency department by the general practitioner, which affected the answers of the CDSS questionnaire. Further, as in real life, some diagnoses were not accurately recorded within the electronic health records by the physicians in the emergency department, although cases with empty diagnoses and cases with a code denoting a Z-diagnosis were excluded.

### Usability

Usability and user interaction have been noted as key elements for evaluating a CDSS [2,29]. In this study, the CDSS (Klinik Access) achieved a mean SUS score of 78.2 (SD 16.8), which indicates good usability and is in the highest quartile when evaluating the SUS [30]. There is a lack of studies that have evaluated the usability of a CDSS in a real-world setting with a diverse patient sample that actually used the CDSS themselves. In addition, our study population of 247 patients is fairly large when compared to other study populations, considering the real-world setting of our study. Fraser et al [8] showed the acceptable usability of a CDSS that used the Technology Acceptance Model among 40 emergency department patients. Additionally, in a study consisting of 49 psychotherapy outpatients, the CDSS (Ada) achieved a mean SUS score of 81.5 [6]. However, this study was performed in a narrow patient context with a more limited study population.

With regard to gathered usability feedback, the fact that the patients with an acute ongoing condition used the CDSS while waiting for a triage nurse in the emergency department is an important detail to consider, and this activity could have negatively impacted the results. Patients filled in the CDSS web-based form at the emergency department waiting room while experiencing an acute ongoing condition and not, for example, at home without distractions, as in the typical primary care setting process. Although participation was voluntary, according to a few answers, some patients had low motivation to fill in the form, most likely as they were aware that the CDSS would not have affected their actual care in any way. Additionally, some answers in the CDSS questionnaire were clearly inadequate. Certain patients had difficulties with choosing the right location for their symptoms and conditions. Some patients also omitted some of their symptoms in the selection phase but reported those symptoms in the free-text field instead, which left the algorithm with insufficient information. These factors are partly inevitable when evaluating patients in an emergency department setting. However, motivation issues could have affected the results due to the study design, in which the CDSS was separate from the actual care. These limitations underline again the challenges of CDSS studies in a real-world setting compared to those of studies using clinical vignettes.

As the positive version of the SUS questionnaire was added solely for the research purposes, it was collected by using a separate web-based form after patients completed the CDSS form, and it did not include the medical information of the patients. Therefore, we could not evaluate the usability among different user categories, such as age groups or gender. Nevertheless, the mean SUS score describes well the results of the whole study population, as 95.4% (247/259) of the study population completed the SUS questionnaire.

There has been no reported statistical difference between the original and positive versions of the SUS [31]. This study was performed in the emergency department; thus, the ease of answering the questionnaire is relevant for the patients [13]. In addition, the positive version of the SUS has been suggested for users with cognitive load or stress, which is typical in an emergency department setting [31].

### Strengths and Limitations of This Study

There are clear strengths in this study. We want to underline the fact that, unlike the majority of the previous studies that explored CDSSs and symptom checkers (with data provided by the patients themselves), the population sample in this study consisted of real patients who entered the emergency department of a university hospital, and patient CDSS data were entered via the internet by the patients themselves [4,7]. To the authors’ knowledge, this is the first study to assess the diagnostic performance and triage sensitivity of a CDSS in a broad clinical setting, using patient-submitted data. The patient sample is representative of the normal adult patient population entering the emergency department in Finland and is presumably unbiased by demographic variance, which strengthens the findings of this study [20].

This study also has some limitations. Even though this study included a broad set of patients, it excluded children, adolescents, and patients with cognitive impairments. This was due to the fact that these patient groups may need another person to fill in the web-based CDSS form, which would have likely affected the study results. In fact, unlike the majority of CDSS technologies, the CDSS assessed in this study (Klinik Access) does include children and adolescents, and a study is in progress to assess the diagnostic and triage performance of the CDSS for children and adolescents aged <18 years in a university hospital setting. Further, the patient selection could have been biased, as the patients were voluntarily participating in this study. We also excluded patients who arrived to the emergency department via prehospital emergency care (ambulance). Additionally, 49.2% (122/248) of patients were female and 50.8% (126/248) were male in this study, and the mean age was 46 (range 18-82) years. Therefore, the patient population in this study reflects the typical patient sample in a university hospital emergency department. The fact that the evaluations of the assessing physicians were reliant on the recordings of a triage nurse can also be considered as a limitation. One assessing physician experienced challenges in accessing the appropriate electronic health record data for 12 patients, which were due to the access permissions used within the electronic health records of the emergency department.

The patients were informed that this study would not affect their normal assessment or the care of their condition. This could have lowered patients’ motivation to complete the web-based CDSS form, which in turn could have resulted in inadequacies in answers. If true, this would directly diminish the study results. These issues are unavoidable when dealing with real-world patient data and, again, underline the challenges of CDSS studies using real-world data compared to those of vignette studies. To tackle these limitations, further studies should assess health care professionals' practical use of a CDSS to show the performance and possible benefits of the CDSS.

### Conclusions

This study is the first to validate a CDSS with a large, real-world patient population within a university hospital emergency department. The CDSS was found to be safe to use, with no missed urgent cases, and was equally as sensitive as emergency physicians’ judgments in urgent patient cases. It provided acceptable differential diagnoses and good patient usability, as evaluated via a positive version of the SUS questionnaire. The CDSS should further be evaluated for its practical use (eg, studies in which health care professionals can use the CDSS and its output in real time). This would likely further demonstrate the practical benefits and effectiveness of CDSS technologies in emergency medicine.

## Abbreviations

CDSS: clinical decision support system
ESI: Emergency Severity Index
*ICD-10*: *International Statistical Classification of Diseases, Tenth Revision*
SUS: System Usability Scale
